# The Influence of Employee Empowerment on Competitive Advantage in Hospitals within Nairobi, Kenya

**DOI:** 10.24248/eahrj.v5i1.648

**Published:** 2021-06-11

**Authors:** Pamleila N Ntwiga, Maina Muchara, Peter Kiriri

**Affiliations:** a United States International University, Africa

## Abstract

**Background:**

The study examined the influence of implementation of employee empowerment on competitive advantage in hospitals within Nairobi. The study looked at the following aspects of employee empowerment; competence, teamwork, motivation, reward and recognition. Employee empowerment is derived from the Total Quality Management (TQM) principles bringing forth competitive advantage that results from high customer satisfaction levels, employee satisfaction and operations efficiency.

**Methods:**

A descriptive correlational research design that applied positivism philosophy. Data was collected from both private and public hospitals within Nairobi targeting patients who were admitted in these hospitals for more than three days during the study period and senior employees of the respective hospitals. There were 308 participants, 154 hospital employees and 154 patients from 31 hospitals within Nairobi. After institutional and individual consent was obtained, participants filled a self-administered questionnaire. The collected data was coded into SPSS Version 23 software and the analysis was done using descriptive and inferential statistics.

**Results:**

The findings illustrated that employee empowerment significantly predicted competitive advantage. High responsiveness and good attitude, being reliable, empathy and assuring the patients of their state best enhance patients’ and employee satisfaction.

**Conclusion:**

This brings out the importance of realigning the staff inputs towards improving patient experiences, as well as considering employees’ performance as individual instead of considering them as teams.

## BACKGROUND

Employee empowerment involves the application of Total Quality Management (TQM). TQM requires a combination of a set of management principles with the right tools and techniques to enable the employees to carry out those management principles in their day to day operation so as to amount to continuous quality improvement.^1^ Competitive advantage is defined as an organisation's ability to attain market superiority.^2^ Competitive advantage is the core concept of strategic management that every firm seeks to retain. Porter provided a framework that models an industry as being influenced by 5 forces.^2^ Porter advised strategic business managers to work towards developing a competitive advantage over the organisations’ rival. This in turn acknowledges that human capital is a key component of an organisation that leads attaining competitive advantage. This is further echoed by the World Health Organization (WHO) which has championed and supported the growing interest in healthcare quality, through supporting projects that address aspects of quality such as service delivery, training, management and technical guidance and spreading awareness of healthcare quality issues.

Numerous studies have been conducted in Kenya to determine the influence of employee involvement on competitive advantage in other sectors other than the health sector.^3^ There are studies to assess the existence of employee involvement conducted in two private hospitals in Nairobi to assess the performance and effectiveness of the quality systems.^4^ These studies focused on various aspects of TQM within Kenya; however, there is little evidence of research and literature on employee empowerment and its influence on competitive advantage in the Kenyan healthcare sector. This study therefore sought to determine the influence employee empowerment has on competitive advantage within the hospitals in Nairobi.

The study was based on the Deming's Theory of profound knowledge and the Resource Based View (RBV). Total Quality Management (TQM) is a people focused management system that aims at continual increment in competitive advantage at a sustainable, affordable cost and an integral part of high-level strategy that operates horizontally across all departments while ensuring that all employees are involved extending to customer and supply chain.

The Deming theory of profound knowledge is a management philosophy grounded on system theory. The application of such theory within the organisational systems, that lead to learning the implementation processes that contribute to the continuous improvement of the processes, services, products, employee fulfilment that would result to customer satisfaction.^5^ The foundation of TQM is philosophically based on scientific methods, since they involve people, tools, systems, and methods. These systems allow change while the philosophy is unchanged.^2^ Therefore, total quality management is the durable and persistently improving effort by every person in an organisation to understand, meet and surpass the expectation of the customer while involving the employees.

To have satisfied customers, organisations are learning that they must have a satisfied workforce first. Simply put, empowerment means giving employees authority to assess and make decisions on what they consider is right to do, to take control of the day to day work, to take risks and learn from their mistakes.^5^ It is the process of delegating decision-making power and authority. It is noted that employee empowerment is the missing piece of the puzzle that has been occasioned by the growing global competition, institutional restructuring and the importance placed on service quality and customer satisfaction.^6^

While workforce is referring to everyone who is actively involved in accomplishing the work of an organisation. Workforce engagement is defined as the employee commitment both intellectually and emotionally towards accomplishing the organisational work, vision and mission. This is translated to mean that employees find personal motivation in what they do that strengthens the emotional bond to their employment.^7^ Suggesting that the high level of engagement has proved to generate higher levels of employee satisfaction and increases organisational performance. All organisations that have high staff engagement are characterised by high performers who are committed to give the utmost best for the benefit of the customer and organisational success.^2^

Motivation has been defined as the art of creating conditions that allow each employee get the work done at the highest level of performance with maximum efficiency.^8^ This is an individual's reaction to a felt need. Motivational theories are categorised as process, content, and environmental theories. These theories are applied to boost high performance within an organisation.^9^ Observing that if managers are keen on improving workplace performance, they must actively manage the motivational process and change the work environment. Since within motivational theory, there is a positive view of the humanity believing in individual growth potential.

Customer satisfaction is the degree to which a customer responds to fulfilment of goods or services.^10^ In hospitals, patient satisfaction is an indicator obtained to compare the patients’ expectation of products or services with the perceived performance.^11^ This is made possible by conducting surveys that measure the average satisfaction level of the patients and their relatives. This could be through courteousness of the employees, time spent waiting for appointment or diagnostic tests, likelihood of return visits, adverse events and clinical outcomes.^12^ Customer satisfaction has risen as a distinct area of inquiry since the 1970s.^13^ Businesses, both big and small have realised the strategic importance of service quality and customer satisfaction as competition become more intense and global. The accomplishment of customer satisfaction has transformed into a good business practice that businesses strive to achieve.^14^

Teamwork is defined as the cooperation or/and coordination of the members of hospital towards achieving and sustaining quality improvement initiatives.^14^ Employee empowerment is by no mean guarantee of employees' total participation to quality improvement initiatives. While initiating quality improvement agenda, the employee input is critical after all improvement of techniques and modern technology cannot supplement what the employees are capable of doing. Implementation of quality techniques such as quality circles, creation of quality improvement teams, initiating quality hit squads and installation of suggestion teams can be used to foster employee involvement and participation.^15^

The employees in the service sector are involved in both production and selling of products in contrast to employees in the manufacturing who have either the role of production or the role of distribution. Since employees in the service sector, are involved in the operations, marketing the service and are being continuously judged by the customers, therefore, the employees must be in a position to control the services they deliver^16^. In summary, employees have a very critical role within the production and marketing of services because they are simultaneously producing and delivering the services. Therefore, employee involvement within quality improvement initiatives is crucial for TQM implementation, as a means to increasing customer satisfaction.^16^

In a study that looked at the effects of human resource management on the implementation of TQM within high technology organisation and their effects on customer satisfaction.^17^ The study showed that there is significant and positive association between human resource involvement TQM and customer satisfaction. This eventually translated to higher competitiveness of the firm. Hence agreement that human resource management involvement has a direct and positive relationship with competitive advantage.^23^

In another study that looked at the link of appropriate human resource management and customer satisfaction in Malaysian small service businesses, it was concluded that human resource involvement did not show any significant effects on customer satisfaction.^18^ One possible reason that contributes to such unconstructive results could be due to the measurement of human resource focus. Human resource focus may refer to teamwork. Too much focus on teams makes the individual employee less responsive to their jobs as they always depend on team performance.^19^

The value of gratifying the psychological needs of the employees. This they argued that just as customers seek services with expectations, the employees too, join the organisation with psychological needs. Therefore, only when the employees are satisfied with the organisations ability to meet their needs, are they likely to fulfil the expectations of the customers.^20^ Humans direct their abilities, vitalities and competencies largely toward the rewards they value as precious. Hence, leaders must ascertain that rewards promote those actions that enhance the concurrent achievement of multiple organisational preferences. Rewards are defined as those items that enable the employee to feel recognised and appreciated such as motor vehicles or decent office space, not just pay and promotion.^6^

In another study to examine the effects of human resource involvement on client satisfaction in the nursing and care industry. 8 human resource management activities were tested during the study, namely personal development plan, job-related training, annual performance review, employee involvement, protocol for labour-shortage, predictable work schedules, transparent management style, and supportive management style. Contrariwise, employee satisfaction was significantly related to the unit manager style of management and to some extent it had an effect on performance reviews and predictable work schedules.^21^ Another study revealed the association between attitudes of the employee, customer satisfaction and the performance of the department, concluding that the magnitudes of employee attitudes, specifically participation of the team and attention to performance evaluation had positive association with customer satisfaction.^22^ In another study, it was observed that the act of training and development of employees has significant and positive association with financial operational performance, employee performance, and customer satisfaction^23^. This concluded that resource allocation for organisations to train on quality initiative agenda pays off, as professional employees understand various quality implementation tools and concepts of quality, making it possible to positively influence the structure and processes of the organisation. Moreover, when employees are treated as a valuable resource there is a chance of increasing their loyalty to the organisation, increases their motivation level as they take pride in the jobs they do.^23^ These attributes leads to improved performance on employees’ jobs, reduced absenteeism, and lower employee turnover. When employees are educated they will improve quality, reliability, and timeliness in delivering the products or services, this will directly and positively influence customer satisfaction of the organisation^16^.

Furthermore, effective training on quality also increases employees’ skills to work effectively and efficiently, hence reducing complaints and increasing customer satisfaction.^24^ Employee satisfaction is a state fulfilling or exceeding the employees’ emotional state that arises from the positive feedback of job performance and its’ effects on the organisational outcome. Employee satisfaction is acknowledged as an employee's assessment of the overall quality of his or her current job assignment and the effects that result from positively impacting on the organisational input.^25^

In a study on the nature of the service quality and satisfaction relationship, it was observed that there is a positive relationship between appraisal of job performance and the service of employees’ delivered to their customers. ^22^ This indicates the need to have a satisfied workforce in order to deliver quality services to the customer. In a study set out to explore those factors that influence employee satisfaction, it was observed that the main factors that have positive influence on employee satisfaction were; conducive working environment, equitable wage structures, quality of supervision and the nature of work.^26^ Supervisory is further expounded as support, impartiality and autonomy, image of the company, connection and employee development affect employee satisfaction.^20^ Staff involvement, motivation to carry out assigned tasks, employee learning and salary components such as stability of the job and wage complements contribute to the levels of employee satisfaction.^6^ Employees also consider organisations that support training and career development as good to work for.^27^ Finally it was noted that work life balance plays a very important role in employee satisfaction. Thus, when employees perceive these stated requirements as met, consequently, the level of employee satisfaction is enhanced.

From a practical point of view, motivated and satisfied employees are more likely to remain within an organisation since they perceive themselves as gaining higher benefits through continuing to work for such organisations.^25^ Additionally, satisfied employees are likely to provide better services to a firm's customers and improve its performance resulting to firms competitive edge^20^. For this reason, the employees of an organisation have an important role in providing service and should be considered as strategic partners by the leadership in order to deliver quality services, retain satisfied employees and customer that improve the organisation's competitive advantage.^28^

## METHODS

### Study Design and Setting

The study was a mixed research design conducted among the employees and patients of hospitals within Nairobi from whom data was collected through self-administered questionnaire to establish the influence employee engagement has on competitive advantage.

### Study Population

The target population was the hospital employees and patients of level 4, 5 and 6 public and private hospitals within Nairobi.

### Sampling Technique

A census of all 46 registered level 4, 5 and level 6 hospitals in Nairobi was done. Simple random sampling was used to select hospital employees while Stratified random sampling was used to select the patients from the same hospital.

### Data Collection

Data was obtained from the participants using self-administered questionnaires with both open ended and closed ended questions. The close-ended questions allowed the respondent to choose from a list of pre-determined options/answers. That is to say, the respondents were asked to select from a list of options provided on a 5-point Likert scale 1; (strongly disagree), 2(disagree), 3(Neutral), 4 (Agree) and 5 (strongly agree). The questionnaire for hospital employees was used to test the variability of every independent variable and consisted of 3 parts one (1), two (2) and three (3). Part one (1) consisted of questions on the hospital and employee characteristics while part (2) consisted questions on the study objectives, and part (3) consisted of open questions. The patients’ questionnaire was used to measure their satisfaction levels.

### Data Management

The data was entered into excel, edited, and coded. The was then analysed using both descriptive and inferential statistics. The cleaned data was moved from Microsoft Excel® sheet to the SPSS version 23, data analytics software for processing. Each response was assigned a unique identifier that collaborated with the study variables. Each of the ordinal, nominal and scale variables were entered with numeric expressions to create a platform for executing the different types of analyses. Data was entered into 2 different spread sheets, for descriptive statistics. The data was then cleaned by checking for missing variables. The 2 sets of data (from employees and from patients) were measured independently using descriptive statistics for demographic information. Data from employees tested the variability in the independent variables. Under inferential statistics, the 2 sets of data (employees and patients) were triangulated in SPSS and the relationship between the first set of data compared to the second set of data that measured patient satisfaction (dependent variable). This was achieved by moving the patients’ data to the employee SPSS spread sheet by forming additional variables after the employee variables. Constructs of the independent and dependent variables were then computed for correlation and regression analysis. Of the 46 hospitals, 31 (67.4%) responded. One hundred and fifty-nine (159) questionnaires were administered to the hospital employees out of which 134 (87.3%) were properly filled and returned. Further, 134 out of 159 patient's questionnaires were properly filled and returned representing a response rate of 83.7%.

The data was analysed and results presented in table forms. The study used the factor analysis, descriptive statistics and inferential statistics to analyse and present the results. The descriptive statistics used were frequencies, mean and standard deviation. The inferential statistics used were Correlation Coefficients, Chi-squared, oneway ANOVA, and regression analysis.

The following assumptions were made in order to make it necessary for a successful regression: Normality Test, Linearity Test, Multicollinearity Test and Homoscedasticity test.

Data analysis formula

$$\text{Y}={\text{β}}_{0}+{\text{β}}_{\text{i}}{\text{X}}_{\text{i}}+{\text{ε}}_{\text{i}};$$

Where:

α denotes the y intercept where x is zero;

β_i_, is regression weight attached to the exogenous variables:

ε is the error term.

Y= Competitive Advantage

X_1_ = Employee Empowerment

### Ethical Consideration

The research proposal was first approved by United States International University Africa information policy which concerns itself with the maintenance of ethical standards and protection of research subjects. Approval was sought from National Commission for Science Technology and-Innovation (NACOSTI). Furthermore, permission was obtained from each of the participating hospitals. The researcher weighed the sensitivity of the topic in designing the data collection instruments and determining what was permissible. The participants were asked to give their consent; hence the study was guided by the principle of informed consent. The respondents were free to decide whether to participate or not (voluntary participation). Each respondent was assigned a unique identifier making all the collected data source anonymous.

## RESULTS

The study sought to examine the extent to which employee empowerment influence competitive advantage within the hospitals in Nairobi. Employee empowerment was measured using 4 parameters namely; competence, teamwork, motivation, and rewards and recognition. The measures for competitive advantage were; patient satisfaction, employee satisfaction and operational effectiveness.

### Results for Factor Analysis on Employee Empowerment

The parameter of employee empowerment was measured using fifteen (15) items to produce appropriate measures. Factor analysis was conducted upon the items to ascertain any correlated parameters with the intention of reducing any unnecessary and redundant data. The Kaiser-Meyer-Olkin (KMO) measure of sampling adequacy value associated with employee empowerment was 0.965. The KMO test is used to measure sampling adequacy. When the result shows the KMO value greater than 0.6, this means that the sample is considered adequate. The value for the Barlett's test was x^2^ (136, N= 268) = 1240.499, *p< .05.*

### Results for Descriptive Statistics on Employee Empowerment

The descriptive statistics for employee empowerment are presented in table 1. Percentage (%) distributions, mean (M) and standard deviations (S.D) were the descriptive statistical analysis carried out.

### Correlation between Employee Empowerment and Patients’ Satisfaction

Table 2 illustrates that the constructs for employee empowerment statistically and significantly correlate with competitive advantage. The results revealed that competitive advantage significantly correlated with competence, *r* (268) = 0.454, *p< .05,* and teamwork, *r* (268) = 0.456, *p< .05.* The study also showed that competitive advantage significantly correlated with motivation, *r* (268) = 0.420, *p< .05,* and reward and recognition, *r* (268) = 0.437, *p< .05.* The findings also indicated that employee empowerment significantly correlated with competitive advantage, *r* (268) = 0.453, *p< .05.*

**TABLE 1:** Mean and Standard Deviation for Employee Empowerment

Descriptive Statistics	N	Mean	Std. Deviation
All staff are trained and qualified to perform their duties.	134	3.87	1.088
Managers involve their staff in critical decision making	134	3.40	1.111
The hospital clinical staff's all meet the minimum requirements of the licensing/regulatory bodies.	134	3.78	1.113
Our hospital has the right mix of people and skills to do its work.	134	3.76	1.077
The staff have developed technical skills that make the delivery of their job easy	134	3.75	1.031
The staff exhibit a win-win attitude towards company work	134	3.86	1.005
There is a policy/protocol that encourages and enables our staff to develop their job skills so they can advance in their careers.	134	3.70	1.164
The staff cooperate and work well as a team.	134	3.62	1.082
The staffs are recognised for their work as teams. E.g. “The best performing ward”	134	3.58	1.057
The hospital has a process of awarding high achievers in their field of duty.	134	3.37	1.168
There are training programs for the staff in the field of quality improvement	134	3.44	1.160
The hospital and the managers care about the workforce.	134	3.61	1.182
The staff are committed to the hospital's success.	134	3.39	1.201
The staff performance is evaluated based on the quality of work	134	3.67	1.024
All staff believe our hospital is the best place to work	134	3.61	1.232

**TABLE 2:** Correlation between Measures of Employee Empowerment and Competitive advantage

		Patient Satisfaction	Employee Satisfaction	Efficiency	Competitive Advantage
Competence	Pearson Correlation	.384^**^	.424^**^	.459^**^	.454^**^
	Sig. (2-tailed)	.000	.000	.000	.000
	N	268	268	268	268
Teamwork	Pearson Correlation	.390^**^	.433^**^	.449^**^	.456^**^
	Sig. (2-tailed)	.000	.000	.000	.000
	N	268	268	268	268
Motivation	Pearson Correlation	.351^**^	.394^**^	.428^**^	.420^**^
	Sig. (2-tailed)	.000	.000	.000	.000
	N	268	268	268	268
Reward and Recognition	Pearson Correlation	.361^**^	.412^**^	.445^**^	.437^**^
	Sig. (2-tailed)	.000	.000	.000	.000
	N	268	268	268	268
Employee Empowerment	Pearson Correlation	.381^**^	.426^**^	.457^**^	.453^**^
	Sig. (2-tailed)	.000	.000	.000	.000
	N	268	268	268	268

** Correlation is significant at the 0.01 level (2-tailed)

### Chi-Squared Test on Employee Empowerment

The results in Table 3 showed that there was enough evidence to conclude that there is a statistical significant association between employee empowerment and competitive advantage x^2^ (2730, *N* = 268) = 3848.172*, p < .05.*

**TABLE 3:** Chi-Squared Test on Employee Empowerment

	Employee Empowerment
Pearson Chi-Square	3848.172a
Df	2730
Asymp. Sig. (2-sided)	.000

** Correlation is significant at the 0.05 level (2-tailed).

### One-Way ANOVA on Competitive Advantage for Employee Empowerment

This study sought to conduct a one-way ANOVA test to ascertain whether there were significant differences between the means for competitive advantage for employee empowerment and the demographic variables of this study; age of the respondents, number of hospital beds, and type of hospital. The findings of this study showed that there was significant differences in the means across respondents’ age, *F* (4, 129) = 2.096, *p< .05.* The study also showed that there were no significant differences in the means across respondents’ number of hospital beds, *F* (4, 129) = 0.957, *p> .05,* and type of hospitals, *F* (4, 129) = 1.272,*p> .05.* This is shown in Table 4.

**TABLE 4:** One-Way ANOVA on Competitive Advantage for Employee Empowerment

ANOVA		Sum of Squares	Df	Mean Square	F	Sig.
Age in Years	Between Groups	52.164	4	1.373	2.096	.002
	Within Groups	62.224	129	.655		
	Total	114.388	133			
Number of Hospital beds	Between Groups	59.918	4	1.577	.957	.548
	Within Groups	156.478	129	1.647		
	Total	216.396	133			
Type of hospital	Between Groups	9.795	4	.258	1.272	.175
	Within Groups	19.257	129	.203		
	Total	29.052	133			

** Correlation is significant at the 0.05 level (2-tailed)

### Regression Analysis and Hypothesis Testing for Employee Empowerment

This study used the regression model to observe whether employee empowerment explained changes in competitive advantage. A variety of assumptions for regressions were carried out before conducting the regression analysis.

### Assumptions for Regression Analysis Employee Empowerment

The below assumptions for linear regression were tested and they all met the assumption criteria.

Linearity: In the scatter plot, the results showed the deviation from linearity, p value above 0.05 Multi-collinearity: Variance Inflation Factors (VIF) of 2.708

Homo-scedasticity: results indicated the value of the Levene Statistic was *F=1.006, P-value 0.186> .05.*

**FIGURE 1.**
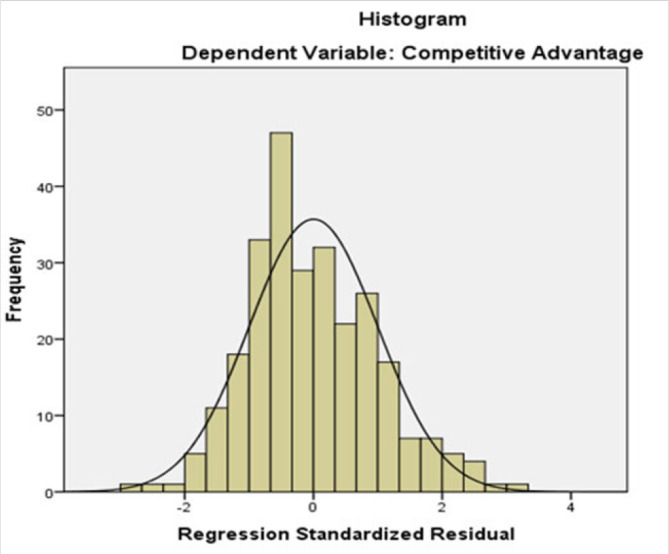
Normality Plot Mean=-3.54E-15 Std. Dev.=0.998 N=268

Normality: Figure 1, depict a histogram of the distribution of the residuals plotted and inspected for the normality test

### Regression Analysis and Hypothesis Testing

A statistical tool that is carried out to examine if one or more independent variables predict the changes in the dependent variable is known as a regression analysis. This study used multiple linear regression analysis to examine the influence of employee empowerment on competitive advantage within the hospitals in Nairobi. This study tested the null hypothesis:

**H_04_**: Employee Empowerment does not have a significant influence on competitive advantage within the hospitals in Nairobi.

### Regression Model Summary

The findings in Table 5 illustrated that employee empowerment explained 22.2% variation in competitive advantage within the hospitals in Nairobi, *R^2^* = 0.222. The results established that 22.2% of the changes in competitive advantage within hospitals in Nairobi could be explained by the employee empowerment.

**TABLE 5:** Regression Model Summary for Employee Empowerment

Model Summary				
Model	R	R Square	Adjusted R Square	Std. Error of the Estimate
1	.471^a^	.222	.210	.28056

a. Predictors: (Constant), Employee Empowerment, Teamwork, Competence, Motivation

### Regression ANOVA

The regression ANOVA shows the variability levels in a regression model and tests the significance of the model. Table 6 presents the results of the regression ANOVA for employee empowerment and competitive advantage. The findings showed that the model was statistically significant in linking employee empowerment with competitive advantage within the hospitals in Nairobi, *F* (5, 262) = 2.615, *p < .05.* According to the results, the influence of employee empowerment on patients’ satisfaction was significant. The model was critical in explaining the relationship and considering the importance of F-statistic.The null hypothesis was rejected.

**TABLE 6:** Regression ANOVA for Employee Empowerment

ANOVA^a^						
Model		Sum of Squares	Df	Mean Square	F	Sig.
1	Regression	5.917	4	1.479	18.792	.000^b^
	Residual	20.702	263	.079		
	Total	26.619	267			

a. Dependent Variable: competitive advantage

b. Predictors: (Constant), Employee Empowerment, Teamwork, Competence, Motivation

**Correlation is significant at the 0.05 level (2 tailed)

### Regression Coefficients

A regression coefficient is a statistical tool that predicts how the dependent variable changes as a result of a unit change in the independent variable. The multiple linear regression was conducted with an aim of determining the magnitude and direction of the relationship between employee empowerment and competitive advantage within the hospitals in Nairobi. The findings of this study are demonstrated in Table 7.

**TABLE 7:** Regression Coefficients for Employee Empowerment

Coefficients^a^						
Model		Un-standardised Coefficients		Standardised Coefficients		
		B	Std. Error	Beta	T	Sig.
1.	(Constant)	1.434	.024		59.900	.000
	Competence	.174	.061	1.087	2.848	.005
	Teamwork	.078	.040	.471	1.942	.053
	Motivation	.205	.075	1.212	2.730	.007
	Reward and Recognition	−.121	.045	−.706	−2.680	.008
	Employee Empowerment	.073	.009	.431	7.792	.000

a. Dependent Variable: Competitive Advantage

The results from this study showed that competence, motivation, and reward and recognition significantly predicted patients’ satisfaction, β = 0.174, *t (268*) = 2.848, *p* <.05, β = 0.205, *t (268*) = 2.7 3 0, *p* <.05, β = −0.121, *t (268*) = −2.680, *p <.05* respectively. The findings of the study showed that teamwork has no significant relationship with competitive advantage, β = 0.078, *t (268*) = 1.942, *p >.05.* From a general point of view, employee empowerment significantly predicted competitive advantage, β = 0.073, *t (268) = 7.792, p <.05.* The implication of the results is that a unit change in employee empowerment would lead to significant increase in competitive advantage within the hospitals in Nairobi by 0.073 units. This study, therefore, concluded that employee empowerment significantly predicted competitive advantage within the hospitals in Nairobi.

### Conclusion of Regression Analysis and Hypothesis Testing

The findings of the multiple linear regression analysis established that employee empowerment positively and significantly predicted competitive advantage within the hospitals in Nairobi, *R^2^* = 0.222, *F* (5, 262) = 2.615, *p < .05;* β = 0.073, *t (268*) = 7.792, *p <.05.* This meant that 22.2 percent of the variance in competitive advantage within the hospital in Nairobi would be explained by employee engagement. The regression model was also found to be statistically significant in predicting the relationship between employee engagement and competitive advantage, *F* (5, 262) = 2.615*, p < .05.*

The multiple linear regression coefficient for employee empowerment implied that every unit change in employee empowerment predicted 0.073 units change in competitive advantage. Pearson value of *p < .05* was adopted by the study and the regression coefficient showed that the p-value of the regression coefficient *(ft*) was *p < .05.* This study, therefore, rejected the null hypothesis and concluded that employee empowerment positively and significantly influences competitive advantage within the hospitals in Nairobi.

## DISCUSSION

The study provides an insight of the influence instituting employee empowerment has on competitive advantage within the hospitals in Nairobi. In general, the findings of this study indicated that; for employee empowerment and competitive advantage, significant differences existed between the means across respondents’ gender, age, number of hospital beds, type of hospitals, and ISO certification. This showed that irrespective of demographic information of the respondents, they perceived private hospital employees as more approachable and responsive than their colleagues in the public sector. This concurred with the findings from a study conducted in Singapore that showed patients who are admitted in private hospitals seem to have higher expectations from their care givers in comparison to those patients who are admitted in public hospitals due to the difference in cost of care incurred by private patients. These hospitals are more concerned about meeting the needs of their customers so as to ensure that their customers (patients) keep coming back to them. ^30^ Furthermore, those few who can afford private hospitals belong to a higher social class and have higher expectations from their caregivers. Further, it is noteworthy to consider that this study was carried out among small hospital in developing country, and the fact that small organisations do not seem to leverage staff role as a strategy tool to aid in improving customer satisfaction but focuses on the delivery of each staffs’ job role^19^. In contrast, in another study it was observed that human resource focus correlates positively with customer satisfaction.^24^

This study suggests that, employee empowerment characteristics such as motivation constitute a positive factor in improving competitive advantage. This is in agreement with other studies that showed that enhancing motivation, impacts on the performance of the organisations and enables them to gain public interest making them more popular.^32^ As observed contrary to manufacturing industry, employees in the service industry are not only linked in the production process but also in the selling of the services, hence, close interaction with the customers that require the staff involvement calls for a vast understanding of quality initiatives of the hospital. The employee results indicated that working in teams did not significantly affect patient satisfaction, on the other hand, patient results indicated that the staff worked well in teams as they handle patients equally and with courtesy at all time. This creates room for further discussion as to whether working in teams really has an impact on patient satisfaction.

In general, the study revealed that there are some high initial positive responses to the questions relating to the facets of employee empowerment through training and competitive advantage. This concurs with another study conducted to determine whether employee empowerment improves organisational effectiveness within South African Universities.^31^ The study revealed that training and development is an important facet of employee empowerment. Information sharing, training and trust seem to be the main contribution to the overall job satisfaction when employees are empowered. Further, agreeing with other studies which also found a positive relationship between employee empowerment and patients’ satisfaction and attributed this to the mode of measurement applied to training and education, since the expectation is that training and education practiced in the service industries are more focused on the practice of the technical skills and not that of the delivery of service by itself. ^16,24^ This is supported by the fact that both private and public hospitals should plan to provide effective training to clinical and nonclinical people to enhance their skills in communication and motivation for them to provide premier services to their patients.^17^ This guides on the emphasis on the need for training programs on patient relationship management as a strategy to enhance employees’ performance that is geared towards impacting patients experiences.

Further, the study found that employee competence had a positive influence on competitive advantage. This finding agreed with the findings of another study which revealed that managers involved their staff in critical decision making.^25^ The author studied the effects of human resource management within the nursing and care delivery industry and observed that employee performance was related primarily to management style of the unit head and the ability to empower the employees to make critical decisions. As good corporate citizens, organisations must work to achieve long-term sustainability for themselves and their customers through a team of well skilled and experienced team.^33^

However, the study revealed that teamwork did not significantly influence competitive advantage. The findings of the study agreed with other studies which found that employee involvement in quality management can take various forms, including: extrinsic involvement, where employees’ participation and contribution to continuous quality improvement is linked with a system of collective TQM oriented rewards, comprising extrinsic monetary and/or nonmonetary rewards and recognition. These results however contradict another study that attributed these findings to measurement of employee involvement that refers to teamwork that could lead to individuals being less responsive as the job outcomes are dependent of team performance^19,32^. However, the findings of this study indicated that these factors had insignificant influence on patient satisfaction. This could be explained by the fact that majority of the staff respondent indicated that employees were recognised for their work as teams that is to say, as the best performing ward as opposed to the best performing staff member. This could have created a sense of lack of individual appreciation even when the employee understood the job role and exhibited competence in performing such duties.

The study also found that reward and recognition significantly predicted competitive advantage. Indicating that employee perceptions of reward and recognition include not only pay and other financial rewards, but also procedural justice in the form of performance appraisal systems, career and promotion opportunities, superior subordinate relations, and job assignments. Therefore, when dealing with employees within service industries such as hospitals, the procedural facades of organisational reward systems requires greater sensitivity on the part of the management while dealing with reward and recognition issues.

This is so since employees consider fair treatment at work as not merely good pay but also other aspects such as genuine and timely performance appraisal, recognition, supervisor-employee relationship, career development, and promotion opportunities.^6^-^8,13^ These aspects of employee rewards to a greater extend, determines the effectiveness of an organisation especially in terms of the customer satisfaction levels. However, another study conducted in South Africa indicated all aspects of employee empowerment except rewards also portray moderately high scores that could result to higher performance of an organisation.^31^

### Strengths and Limitation of the Study

The findings of the study offer a number of insightful observations that healthcare providers could utilise to differentiate and/or compare the outcome of service (patient satisfaction, employee satisfaction and operations efficiency) from competitors, as an important tool for assessing the levels of competitive advantage in respect to the quality of healthcare services delivered by the competitors while implementing the Total Quality Management principles.

The scope of the study was limited to hospitals within Nairobi. We suggest that further studies be carried out within other industries, sectors and geographical area.

## CONCLUSION

The findings of this study provides a strong signal to the hospital leaders that the practice of empowerment at work is important in bringing satisfaction to both the employees and the patients hence improving their hospitals’ competitive edge. The study concluded that the best performing employees are those that are well trained and qualified to perform their duties. Through training, staff gain technical skills that make the delivery of their job easy and enhance competitive advantage levels. From a general point of view, the study concluded that when employees are competent, they work as a team, they are motivated andthus leading to enhanced competitive advantage.

With the purpose of improving competitive advantage of the hospital, policy makers and the regulating agencies could utilise the study findings to develop policies and guidelines that help to improve and sustain the high-level quality services through empowered employees. The information generated from the study could be used for public education so that the patients are able to make informed decision on the choice of hospitals to go to.
